# Deformation of and Interfacial Stress Transfer in Ti_3_C_2_ MXene–Polymer Composites

**DOI:** 10.1021/acsami.1c21611

**Published:** 2022-02-21

**Authors:** Mufeng Liu, Yuling Zhuo, Asia Sarycheva, Yury Gogotsi, Mark A. Bissett, Robert J. Young, Ian A. Kinloch

**Affiliations:** † National Graphene Institute, Henry Royce Institute and Department of Materials, School of Natural Sciences, The University of Manchester, Oxford Road, Manchester M13 9PL, U.K.; ‡ A. J. Drexel Nanomaterials Institute, and Department of Materials Science and Engineering, Drexel University, Philadelphia, Pennsylvania 19104, United States

**Keywords:** MXenes, 2D nanomaterials, micromechanics, *in situ* Raman, mechanical properties, stress transfer, nanocomposites

## Abstract

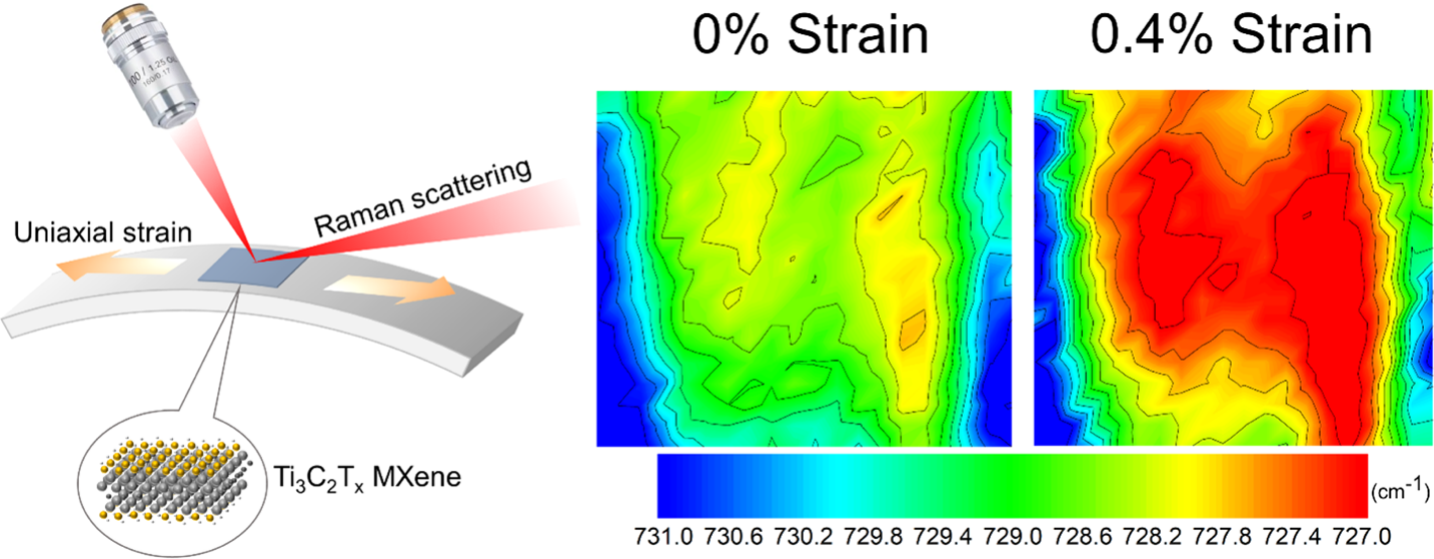

Transitional metal carbides and nitrides (MXenes) have promise for incorporation into multifunctional composites due to their high electrical conductivity and excellent mechanical and tribological properties. It is unclear, however, to what extent MXenes are also able to improve the mechanical properties of the composites and, if so, what would be the optimal flake size and morphology. Herein, Ti_3_C_2_T*_x_* MXene is demonstrated to be indeed a good candidate for mechanical reinforcement in polymer matrices. In the present work, the strain-induced Raman band shifts of mono-/few-/multilayer MXenes flakes have been used to study the mechanical properties of MXene and the interlayer/interfacial stress transfer on a polymer substrate. The mechanical performance of MXene was found to be less dependent upon flake thickness compared to that of graphene. This enables Ti_3_C_2_T*_x_* MXene to offer an efficient mechanical reinforcement to a polymer matrix with a flake length of >10 μm and a thickness of 10s of nanometers. Therefore, the degree of exfoliation of MXenes is not as demanding as other two-dimensional (2D) materials for the purpose of mechanical enhancement in polymers. In addition, the active surface chemistry of MXene facilitates possible functionalization to enable a stronger interface with polymers for applications, such as strain engineering and mechanical enhancement, and in materials including membranes, coatings, and textiles.

## Introduction

MXenes have been the focus on intense research over the last decade^1,2^ mainly due to their extraordinary electronic properties and electrochemical performance.^3−7^ They comprise a large family of more than 100 stoichiometric two-dimensional (2D) materials that have been either prepared experimentally or predicted theoretically to exist and a virtually infinite number of solid solutions on M and/or X sites.^8^ To date, the most widely studied MXene is Ti_3_C_2_T*_x_*, which is typically prepared by the selective etching of Al from the MAX phase Ti_3_AlC_2_.^9^ The commonly used HF-containing etchants introduce −O, −OH, and −F terminating groups that are represented by T*_x_* in the Ti_3_C_2_T*_x_* formula.^9^ One route to translate the promise of MXenes to the macroscale is to use them in conjunction with polymers,^10^ such as for polymer-based electromagnetic interface (EMI) shielding coatings,^11−13^ dielectric permittivity,^14,15^ sensors,^16^ light-emitting diodes,^17^ coatings with good tribological properties,^18,19^ conducting polymer-based supercapacitors^20−22^ and polypyrrole (PPy)/Ti_3_C_2_T*_x_* composites for electrodes.^23^ All of these applications require control of the MXene/polymer interface, which is still poorly understood. So far, the only micromechanical analysis of interfaces has been conducted using simulation methods^24^ and no experimental studies have been undertaken regarding the deformation and interfacial stress transfer for either monolayer or multilayer MXenes in polymers. Thus, it is unclear how to optimize the design of MXene composites so that they have good mechanical properties, in addition to their outstanding electrical and tribological properties.

A limited number of studies have been undertaken on the mechanical properties of MXenes. Atomic force microscope (AFM)/nanoindentation on suspended small single-layer and double-layer MXene flakes has found that a monolayer Ti_3_C_2_T*_x_* possesses a thickness of ∼1 nm, a Young’s modulus of ∼330 GPa, and a tensile strength of ∼17 GPa.^25^ Nevertheless, the detailed mechanical performance of multilayer and “imperfect” MXenes had not been reported until recently, when Firestein et al.^26^ conducted a nanoscale *in situ* tensile test in a transmission electron microscope (TEM). It was revealed that multilayer MXenes (∼40 to ∼100 nm thick) displayed strengths of up to 600 MPa and moduli up to ∼200 GPa, both of which are significantly lower than the values measured for monolayers.^25^ This inferior mechanical performance was attributed to an increasing number of defects and more incomplete layers with increasing thickness of MXene nanosheets.

*In situ* deformation of 2D materials in a Raman spectrometer is an alternative technique to understand both the mechanical properties of individual flakes in real conditions and allow material interfaces to be probed. This technique has been used previously to understand the mechanical performance of other 2D materials in polymers, including graphene,^27−39^ tungsten disulfide (WS_2_),^40−43^ and quite recently hexagonal boron nitride (hBN).^39,44−47^ Typically, the 2D nanomaterials are either deposited on the polymer surface or incorporated into a composite layer, and their deformation is monitored using Raman spectroscopy while uniaxial strain is applied on the polymer substrate *in situ*.^29,34,40,44^ The strain-induced shift of Raman bands from the 2D flake allows the local strain in the flake to be mapped with approximately 1 μm spatial resolution, from which the micromechanics can be studied by the application of analytical models. These studies have shown that the reinforcement by a 2D nanomaterial depends as much on its morphology and surface chemistry as its intrinsic mechanical properties.^27,29−34,44^

In the present study, we have investigated the interfacial stress transfer from a polymer matrix to Ti_3_C_2_T*_x_* MXene using the strain-induced Raman band shift measurements. The use of Raman spectroscopy has allowed us to determine the Raman band shifts induced by uniaxial strain and subsequently to map out strain distributions of MXene flakes during deformation of the substrate.

## Experimental Methods

### Preparation of MXenes

The materials used in this study were synthesized by selective etching of Al layers from Ti_3_AlC_2_. One gram of Ti_3_AlC_2_ powder (<40 μm particle size) was gradually added to the solution of a mixture of hydrochloric acid, HCl, and hydrofluoric acid, HF (Millipore-Sigma) as described elsewhere.^48^ The mixture was stirred for 24 h at 35 °C. After etching, the mixture was washed with deionized water five times by centrifugation in two 150 mL plastic centrifuge tubes at 3500 rpm (2450 rcf) for 2 min until the pH of the supernatant reached 6–7. To delaminate the produced MXene, the sediment from the last centrifugation step was added to a 20% by weight solution of lithium chloride (LiCl) in water at room temperature. The mixture was dispersed by manual shaking and stirred overnight to achieve intercalation. After that, the MXene was washed three times until the supernatant became dark, which is an indication of delamination. Then, the mixture was centrifugated for 1 h at 2450 rcf and the sediment was dispersed in DI water. The residual MAX phase was separated at the last step by centrifugation at 1000 rpm (200 rcf) for 5 min. The remaining supernatant is a dispersion of MXene flakes.

### MXene Flake Characterization

A solution of the exfoliated Ti_3_C_2_T*_x_* MXene flakes in water was deposited onto PMMA beams (Solaris S000, Panelgraphic) either as prepared or diluted by isopropyl alcohol (IPA) (1:1 by volume). Afterward, the beams were dried at room temperature in the air for 1–2 days. The thicknesses of a number of the MXene flakes were examined using a JPK NanoWizard atomic force microscope (AFM) with the QI mode being employed. For the sample top coated with PMMA, the coating procedure took place using spin coating with 4% PMMA in anisole at 500 rpm for 1 min.

The specimens were then placed in a four-point bending rig and the strain applied step wise. The *in situ* bending measurements were conducted on the microscope stage of a Raman spectrometer (laser λ = 785 nm, Renishaw InVia) where the incident laser was perpendicular to the specimen. The laser power was kept low (1%) and the exposure time was 10 s, with or without a number of accumulations. It should be emphasized that a focused laser spot at 100× with power up to 10% could induce thermal oxidation of the flake being measured. The mapping experiments were undertaken using an automated stage with a step size between 0.5 and 1 μm. Optical images of graphene flakes were obtained using the Wire 3 software and an objective of 100× in the Raman spectrometer microscope. The shift of the Raman A_1g_ (∼730 cm^–1^) band in the specimens was studied following the application of strain on the PMMA beam. The strain of PMMA substrates was determined using a resistance strain gauge. All of the MXene spectra were fitted with a single Lorentzian curve, while the baseline of all of the spectra was set to float with the spectra fitted.

## Results and Discussion

### Determination of MXene Flake Thickness

**Figure 1:**
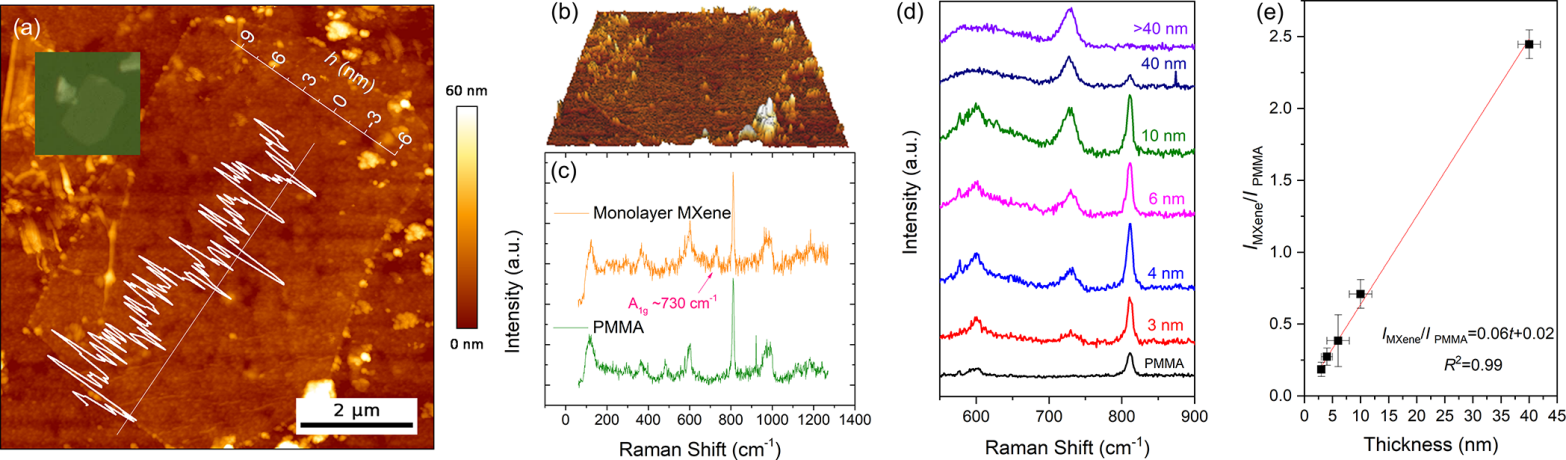
(a) AFM image of a monolayer on the surface of a PMMA beam showing a thickness of 3 nm; the inset shows the corresponding optical micrograph of the flake. (b) 3D view of the same flake indicating a homogeneous thickness throughout the flake surface. (c) Raman spectra of pure PMMA and the monolayer MXene on the PMMA substrate, where the A_1g_ peak of MXene at ∼730 cm^–1^ can be clearly seen. (d) Raman spectra of MXene flakes with different thicknesses confirmed by AFM measurements. The detailed AFM measurements and the full range Raman spectra can be seen in the Supporting Information (SI). (e) *I*_MXene_/*I*_PMMA_ as a function of the thicknesses of the MXene flakes measured by AFM, showing a linear relationship. The parameters *I*_MXene_ and *I*_PMMA_ are the intensities of the Raman A_1g_ peak of the MXene at 730 cm^–1^ and the C=O Raman stretching band of the PMMA at 810 cm^–1^.

The morphology of a Ti_3_C_2_T*_x_* flake on PMMA was examined by AFM, as shown in Figure 1a. The corresponding optical micrograph of the flake is shown in Figure 1a (inset). The AFM height profile of the flake shows a thickness of ∼3 nm, which is an indication of a monolayer,^25,49^ even though the nominal thickness of a monolayer MXene is ∼1 nm.^50,51^ The increased height is attributed to the presence of surface adsorbates that are trapped under the monolayer flake,^49^ a phenomenon found previously for other 2D materials.^52−55^ It can be seen from the three-dimensional (3D) view of the AFM image in Figure 1b that the thickness across the MXene monolayer is homogeneous, indicating complete exfoliation.

In contrast to the monolayer flake measured in Figure 1a,b, representative “imperfect” MXene flakes on PMMA substrates are shown in Figure S1 and described in the Supporting Information, SI. It can be seen that flakes with defective morphologies, including folds, partial restacking, and/or incomplete exfoliation, were also present on the substrate. These morphologies of 2D materials can result in unexpected complexity in mechanical performance^26^ and interfacial stress transfer.^32,34^ Therefore, in this study, such MXene flakes were avoided for the micromechanical characterization.

The Raman spectra are presented for both the monolayer MXene on PMMA and the reference PMMA in Figure 1c. The only MXene band that can be clearly distinguished from the PMMA spectrum is the A_1g_ band at ∼730 cm^–1^, which is a result of the out-of-plane vibration of carbon atoms.^56,57^ A strong peak can be identified at ∼810 cm^–1^ for the PMMA, which is assigned to the C=O stretching vibration.^58^

In order that Raman spectroscopy could be used to estimate the thickness of flakes being studied, the ratio of the intensity of the A_1g_ peak from the MXene (*I*_MXene_) and that of the C=O band of the PMMA (*I*_PMMA_) was calibrated against the thickness of MXene flakes measured by AFM. The AFM measurements were undertaken on five flakes with different sizes and thicknesses that are shown in Figures 1a and S2. These flakes were then located under the optical microscope of the spectrometer and their Raman spectra were obtained (Figures 1d and S2e for full spectral range: 50–1250 cm^–1^). It was found that *I*_MXene_/*I*_PMMA_ increased with the increasing MXene flake thickness. When the MXene was thicker than ∼40 nm, however, the PMMA peak was absent from the spectra due to the incident light being unable to penetrate the MXene and interact with the PMMA underneath. The ratio *I*_MXene_/*I*_PMMA_ was found to have a linear relationship with the AFM measured thicknesses, *t*, in Figure 1e, where *I*_MXene_/*I*_PMMA_ = 0.06*t* + 0.02, for a thickness *t* in nm, with an *R*^2^ coefficient of 0.99. It should be noted that this method of thickness measurement using Raman spectroscopy is not universal and requires calibration for the particular spectrometer being used as the measured intensities can vary with the optical characteristics of the instrument. It should be pointed out that the top coating of the thin layer of the PMMA did not affect the intensity ratio in our experience. We have shown a spectrum of a coated thick flake (no. 5 in Table 1) in Figure S2f, where the PMMA peak at 810 cm^–1^ was absent. This enabled us to estimate the thickness of the flakes that were top coated.

**Table 1:** Raman Band Shift Rates Measured at the Center of the Flakes^a^

#	dω_A1g_/dε (cm^–1^/%)	strain up to (%)	top coating	thickness (nm)/method	lateral size (μm)	γ_A1g_
1	–3.6 ± 0.9	0.5	coated	10 (*I*_A1g_/*I*_PMMA_)	8	0.76
2	–2.4 ± 0.2	0.5	coated	25 (*I*_A1g_/*I*_PMMA_)	8	0.51
3	–1.4 ± 0.7	0.5	coated	>40 (*I*_A1g_/*I*_PMMA_)	6	0.29
4	–3.3 ± 0.9	0.5	coated	15 (*I*_A1g_/*I*_PMMA_)	5	0.70
5	–3.7 ± 0.5	0.6	coated	>40 (*I*_A1g_/*I*_PMMA_)	19	0.78
6	0.1 ± 0.3	(no shift)	coated	>40 (*I*_A1g_/*I*_PMMA_)	30	
7	–3.6	0.4 (only)	coated	>40 (*I*_A1g_/*I*_PMMA_)	19	0.76
8	–3.3 ± 0.5	0.6	uncoated	6 (AFM)	5	0.70
9	–3.7 ± 0.6	0.6	uncoated	3 (*I*_A1g_/*I*_PMMA_)	5	0.78
10	–2.8 ± 0.8	0.6	uncoated	4 (*I*_A1g_/*I*_PMMA_)	3	0.59
11	–2.5 ± 0.3	0.6	uncoated	10 (AFM)	5.5	0.63
12	–3.4 ± 0.3	0.6	uncoated	4 (*I*_A1g_/*I*_PMMA_)	4	0.72
13	–3.7 ± 0.2	0.4	uncoated	3 (*I*_A1g_/*I*_PMMA_)	8.5	0.78
14	–3.7 ± 0.3	0.4	uncoated	3 (AFM)	8	0.78

a Nos. 1–7 flakes are top-coated with PMMA and nos. 8–14 are uncoated. The detailed plots of the band shift rate are shown in Figures S3 and S4. The thicknesses of all of the flakes are either estimated from the Raman intensity ratio *I*_MXene_/*I*_PMMA_ or AFM. The three flakes measured by AFM were included in the calibration of the Raman measurements, as shown in Figures 1e and S2. The lateral sizes were determined from optical micrographs and the values of their aspect ratio were calculated (*s* = *l*/*t*). The values of Grüneisen parameter were calculated using eq 1.

### Strain-Induced Raman Band Shifts and Interlayer Stress Transfer

**Figure 2:**
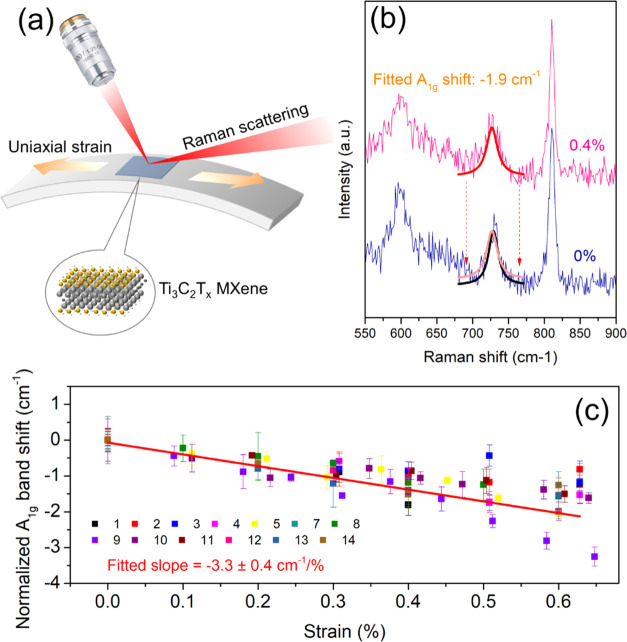
(a) Schematic diagram of the strain-induced *in situ* Raman band shift experiment. The strain on the surface of the deformed beam is uniaxial tension. (b) Raman spectra of the monolayer at 0 and 0.4% strain, showing a band shift of −1.9 cm^–1^/% at the A_1g_ peak. The peak was fitted using a Lorentzian curve and the strain was determined by a resistance strain gauge. (c) Strain-induced Raman band shift rate for all of the flakes with a range of different lateral sizes and thicknesses. The average value of the band shift rate was found to be 3.3 cm^–1^/%. The color coding was based on the no. of the flakes shown in Table 1.

Changes of the Raman band position with strain in the center of MXene flakes were measured *in situ*, as shown in the schematic diagram in Figure 2a. The strain was applied to the PMMA beam using a four-point bending rig, and the spectra at 0 and 0.4% strain for the monolayer flake in Figure 1a are shown in Figure 2b. It can be seen that the position of the A_1g_ Raman band of the MXene undergoes a downshift, as a result of the stress being transferred from the matrix to the flake. The band positions were obtained by Lorentzian curve fitting, and the downshift of the A_1g_ band was found to be around −1.9 cm^–1^ at a strain of 0.4%.

We performed similar measurements at the centers of 14 flakes, of which seven flakes were coated (nos. 1–7) with a thin layer of PMMA on the top and the other seven were uncoated (nos. 8–14). The normalized A_1g_ band positions are plotted against the applied strain for the 13 flakes measured in Figure 2c (flake no. 6 showed no band shift) and plotted individually in Figures S3 and S4. The full width at half-maximum (FWHM) of all of the flakes are tabulated in Table S1. As can be seen, the FWHM of the different flakes measured were found to be similar. Regarding flake no. 6, where no band shift was shown, the reason is believed due to the fact that a good interface might not have formed. Although the upper surface can easily be seen under the optical microscope (Figure S3f), the morphology of the flake underneath the surface could be complex, leading to a poor interface. Overall, the A_1g_ band peak positions shifted to lower wavenumbers with strain up to 0.4% with an average band shift rate (dω/dε) of −3.3 cm^–1^/% over this strain range (Figure 2c). However, for higher strains from 0.4 to 0.6%, A_1g_ band positions became scattered, possibly due to interfacial slip at the higher strain. Similar behavior has also been seen during the deformation of thin flakes of other 2D materials.^29,44^ The detailed results including the band shift rate, thickness of flakes, the lateral sizes of flakes, and the calculated Grüneisen parameter for the A_1g_ mode can be seen in Table 1.

### Grüneisen Parameter

The Grüneisen parameter is used to describe how the change in volume of a crystal lattice affects its vibrational properties. In terms of strain engineering of 2D materials, the Grüneisen parameter can be calculated from the Raman band shifts, which reflects the rate of phonon shift under strain. The Grüneisen parameter for the A_1g_ mode can be evaluated from the Raman band shift rate using the equation^59^
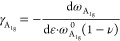
1where (dω_A_1g__/dε) is the strain-induced Raman band shift rate, ω_A_1g__^0^ is the A_1g_ band position (∼730 cm^–1^), and ν is the Poisson’s ratio of the substrate (0.35 for PMMA). The Grüneisen parameter for the A_1g_ mode for uniaxial tension was calculated for all of the flakes measured in this work (listed in Table 1) and gave values mostly in the range between 0.6 and 0.8. If we take an average value of the measured band shift rate (−3.3 ± 0.5 cm^–1^/%), then the Grüneisen parameter, γ_A_1g__, is given by 0.70 ± 0.15.

A previous study^60^ by Zhang and co-workers upon subjecting Ti_3_C_2_T*_x_* MXene to hydrostatic pressure reported a value of γ_A_1g__ of 0.29. They determined the strain by undertaking measurements of the change in lattice parameters of the MXene under pressure and used the out-of-plane strain from the change in *c* lattice parameter for the calculation of the Grüneisen parameter. In our study, we subjected MXene flakes to in-plane strains and obtained a higher value of γ_A_1g__. It should be pointed out that at this stage that molecular crystals do not have a single value of γ_A_1g__. In fact, the Grüneisen parameter for molecular crystals should be represented as a tensor, in a similar way to three-dimensional stresses and strains.^61,62^ This means that there are different components of γ_A_1g__, one for out-of-plane strains, determined by Zhang et al.,^60^ and another for in-plane strains, determined in this presented study. Fortunately, Zhang et al. also measured the in-plane strains from the change in the *a* lattice parameter under pressure, which they found to be more than 3 times lower than the changes in the *c* lattice parameter. This leads to a value of γ_A_1g__ for in-plane strains of more than 3 × 0.29 ≈ 0.9 for their pressurization study,^60^ close to our value of 0.70 ± 0.15 determined from the present investigation by straining Ti_3_C_2_T*_x_* MXene on a substrate.

### Stress Transfer and Interfacial Shear Strength

**Figure 3:**
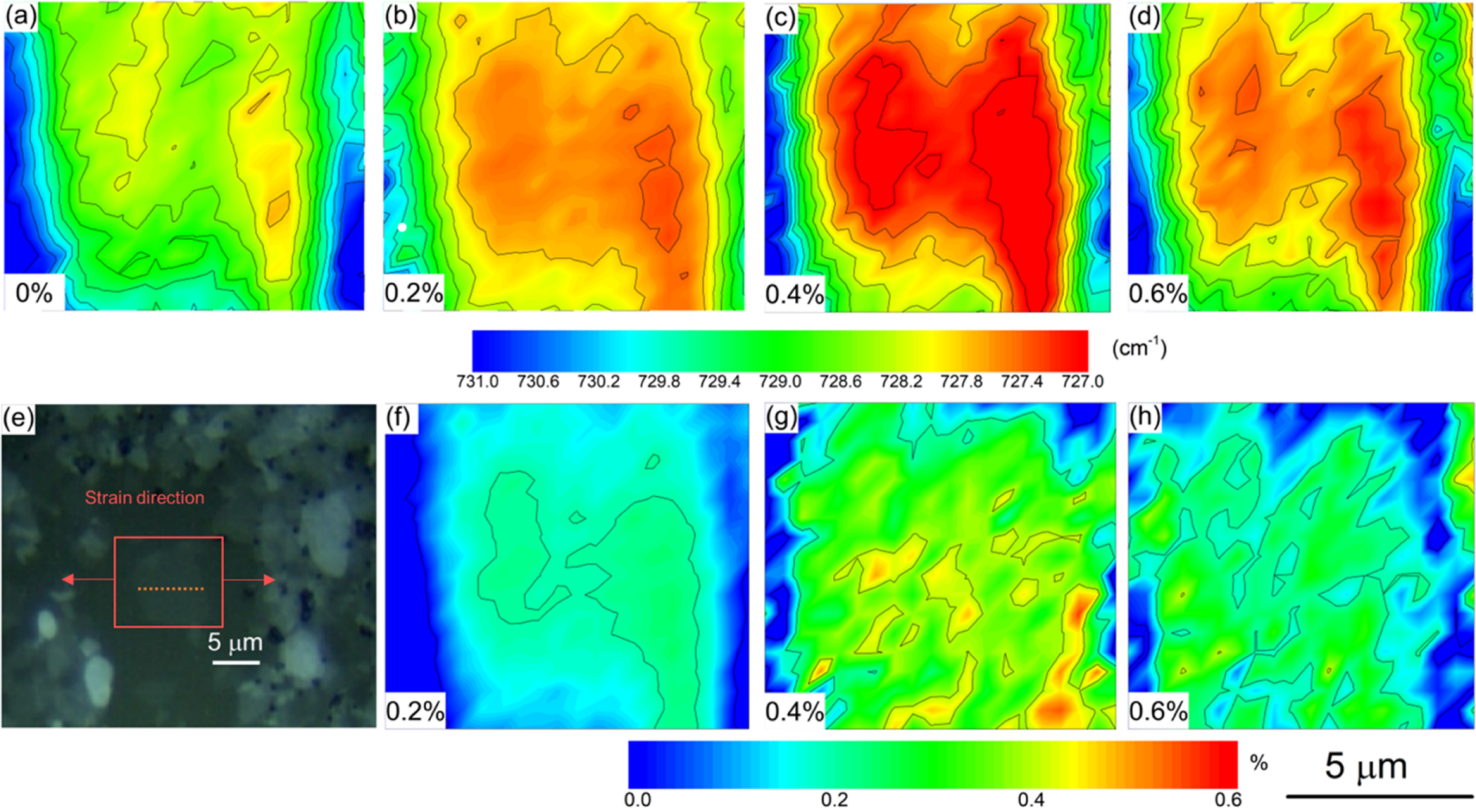
(a–d) Contour maps of A_1g_ band positions at strains of 0, 0.2, 0.4, and 0.6% for a monolayer MXene; (e) optical micrograph of the measured flake; the strain direction is indicated by the arrows, while the dashed line indicates the line mapping positions corresponding to Figure 4a,b; and (f–h) strain distribution at 0.2, 0.4, and 0.6% strains obtained by subtraction of band position mappings at 0.2, 0.4, and 0.6% to that of 0% based on eq 2. (This flake is no. 13 in Table 1.).

By introducing our previous approach established for graphene,^27,29^ it was also possible to monitor the stress transfer from the PMMA substrate to the MXene by mapping the strain distribution over a MXene flake. Herein, we focus on the two monolayers (flake nos. 13 and 14) for the analysis of interfacial stress transfer and the determination of the interfacial shear strength. As can be seen from Figure 3a–d, the contour maps of band positions mirror the profile of the flake, the optical micrograph of which is highlighted in Figure 3e. At 0% strain, the Raman wavenumbers of the A_1g_ band were at ∼729 cm^–1^ for most of the area of the flake. However, some areas in the central part of the flake displayed wavenumbers down to ∼728 cm^–1^, possibly due to variations in terminating groups that affect the vibration frequency of the radiated light slightly.^56^ With increasing strain applied to the PMMA beam, the band positions of the flake central area shifted gradually to lower wavenumbers and finally down to around 727 cm^–1^ at a strain of 0.4%. The direction of the applied strain is indicated in Figure 3e. When the strain was increased to 0.6%, however, the A_1g_ band did not undergo any further downshift relative to 0.4% strain, indicating possible interfacial slippage.^63^ To convert band position mappings into the strain distribution within the flake, we normalized the band positions of 0.2, 0.4, and 0.6% strains to that of 0% strain by subtraction of the band positions at 0% strain. The strain of the MXene at a given location, ε_MXene_, is given by
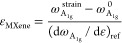
2where ω_A_1g__^strain^ is the band position of the A_1g_ band at the strain of the measurement, ω_A_1g__^0^ is the band position of A_1g_ at the strain of 0%, and (dω_A_1g__/dε)_ref_ is the reference band shift rate (−3.7 cm^–1^/% for flake no. 13 in Table 1). Hence, strain distributions across the MXene flake were obtained at 0.2, 0.4, and 0.6% and are shown in Figure 3f–h. It can be seen that the strain of the MXene flake builds up from the edges of the flake (left and right in the figures) and reaches a plateau value at the central area of the flake (with some scatter of the data).

**Figure 4:**
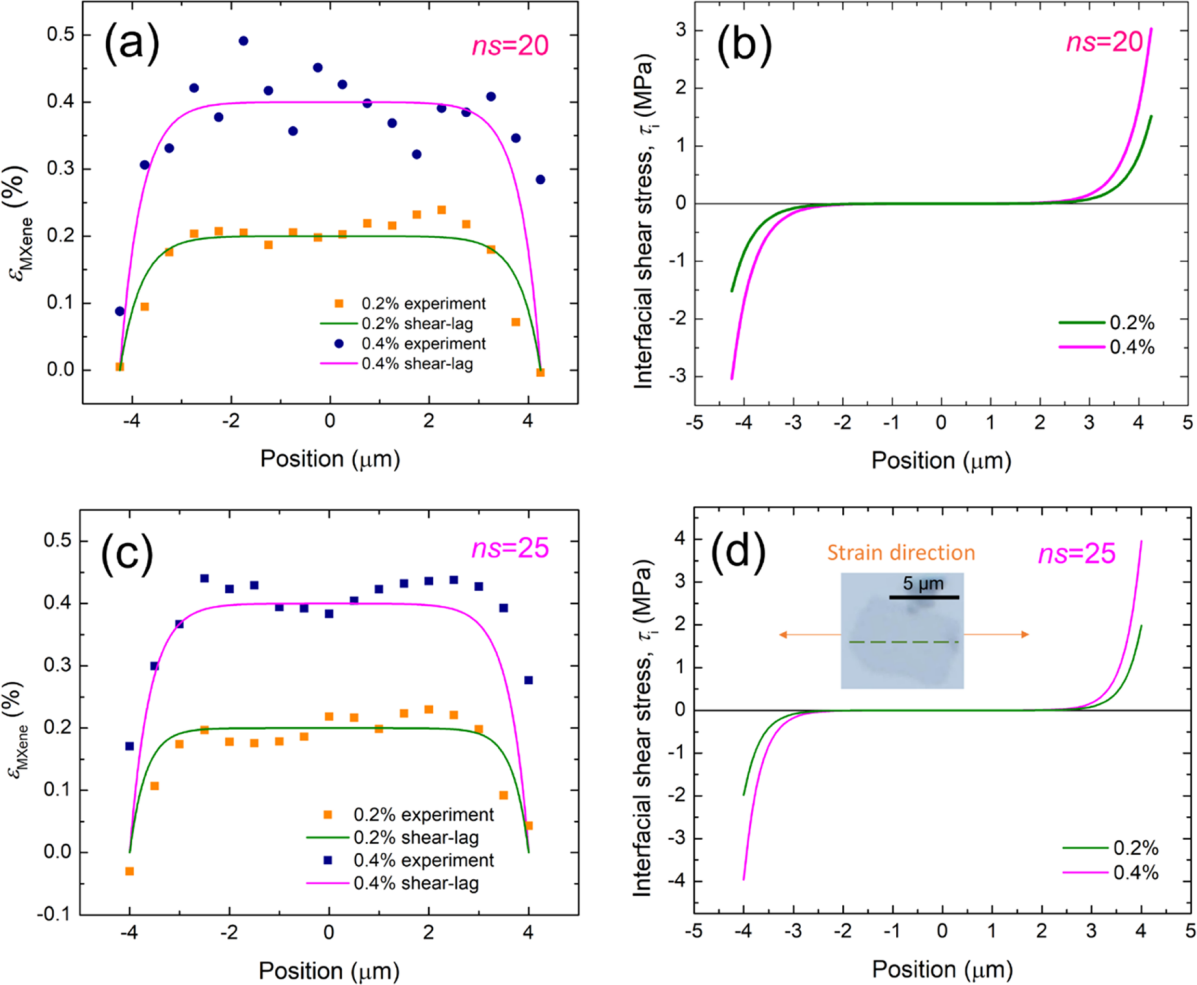
(a) Strain distribution of the MXene flake no. 13 at 0.2 and 0.4% matrix strain; the data points are fitted by shear-lag theory (eq 3); and the *ns* value obtained is 20. (b) Interfacial shear stress against the position at 0.2 and 0.4% strains based on eq 4; the maximum interfacial shear stresses at the flake ends at 0.2 and 0.4% strain were found to be 1.5 and 3.0 MPa, respectively. (c) Strain distribution of the MXene flake no. 14, at 0.2% and 0.4% strain of the matrix; the data points are fitted by shear-lag theory (eq 3); and the *ns* value obtained is 25. (d) Interfacial shear stress against positions of the measurement at 0.2 and 0.4% strain based on eq 4; the maximum interfacial shear stress at the flake ends were obtained to be 2.0 and 4.0 MPa, respectively.

We were then able to analyze the stress transfer efficiency using the shear-lag theory adapted for 2D materials.^27,29,30,32,40,44^ The detailed strain distributions along the axial direction at 0.2 and 0.4% are plotted in Figure 4a as a function of position (shown by the dashed line in Figure 3e). The strain along the MXene flake (ε_M_) can be fitted by the shear-lag theory and is given by^29^
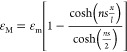
3where ε_m_ is the strain of the matrix; , *s* and *l* are the aspect ratio and length of the flake; *t* is the thickness of the flake (∼1 nm for monolayer)^25^ and *T* is the thickness of the matrix surrounding the flake; *G*_m_ and *E*_M_ are the shear modulus of the matrix and the effective modulus of the MXene flake; and *x* refers to the axial position along the flake. It can be seen in Figure 4a that the experimental data can be fitted to eq 3 using an *ns* value of 20, which gives good agreement with the experimental data at both 0.2 and 0.4% strain. Another monolayer was also examined using the same method (shown in Figure 4c), and this gave an *ns* value of 25. It is known that a higher *ns* value represents better interfacial stress transfer from polymers to the 2D material.^29,30^ Overall, we can conclude that the stress transfer efficiency from the polymer to the monolayer MXene is similar to that for monolayer graphene (*ns* ∼ 20).^29^

With the *ns* values determined, the interfacial shear stress, τ_i_, at the flake ends can now be calculated using^29^
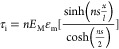
4where *E*_M_ can be taken as the modulus of the Ti_3_C_2_T*_x_* MXene monolayer (330 GPa).^25^ The value of *n* can be obtained from the *ns* value of 20. Using the aspect ratio, *s* (=*l*/*t*), *n* is determined to be 0.002 (flake no. 13 in Table 1), where the thickness *t* is taken as 1 nm for a monolayer. The interfacial shear stress values at the flake ends were estimated from the plots of eq 4 in Figure 4b to be 1.5 and 3.0 MPa for 0.2 and 0.4% strain, respectively. In addition, we performed the same analysis on another monolayer with an *ns* of 25 (Figure 4d and flake no. 14 in Table 1, *n* = 3.1 × 10^–3^) mapped at 0.2 and 0.4% gave an interfacial shear stress of 2.0 and 4.0 MPa, respectively (Figure 4d). Thus, the interfacial shear stress of the monolayer MXene flakes on the PMMA substrate were slightly higher but of similar values compared with those of monolayer graphene at 0.4% (τ_i_ ∼2.3 MPa).

### Reinforcing Efficiency of MXenes

The Raman band shift rates upon applied strains can be correlated with the effective modulus of MXene flakes. The effective modulus corresponds to the reinforcement which the flakes give in the composite and is a function of the intrinsic MXene modulus, interlayer shear between the layers of the flake, length of the flake, orientation of the flake relative to the strain, and the strength of the polymer–flake interface.^27,44^ The band shift data for all of the flakes measured at flake centers have been listed in Table 1, along with their various lateral sizes and thicknesses. It was found that only a minor difference of band shift rates could be found for variations of the MXene thickness measured by AFM from 3 nm up to >40 nm. In contrast, the strain-induced Raman band shift rate of graphene decreases significantly with an increasing number of layers (−52, −53, −44, and −8 cm^–1^/% for mono-, bi-, tri-, and many-layer graphene, respectively).^28^ To examine the interlayer stress transfer and effective modulus of MXenes quantitatively, we adapted our previous approach on the determination of effective modulus of multilayer graphene obtained from Raman band shift^30^ and its correlation to the aspect ratio of the flake. We have demonstrated in Figure 4 that the strain of the flake is a function of the positions of measurement along the flake. At the center of a flake, the highest strain of the flake can be achieved and therefore gives the highest band shift rate along the flake. This maximum band shift rate at the flake center is given by
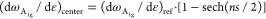
5where the reference A_1g_ band shift for MXene, (dω_A_1g__/dε)_ref_, takes the value of −3.7 cm^–1^/% that was obtained from the monolayers. When the value of *ns* is high (>10), the value of sech(*ns*/2) is close to zero, which indicates that the critical length of stress transfer is achieved, and therefore, the band shift rate at the flake center is the same as the reference A_1g_ band shift for MXene (−3.7 cm^–1^/%).

**Figure 5:**
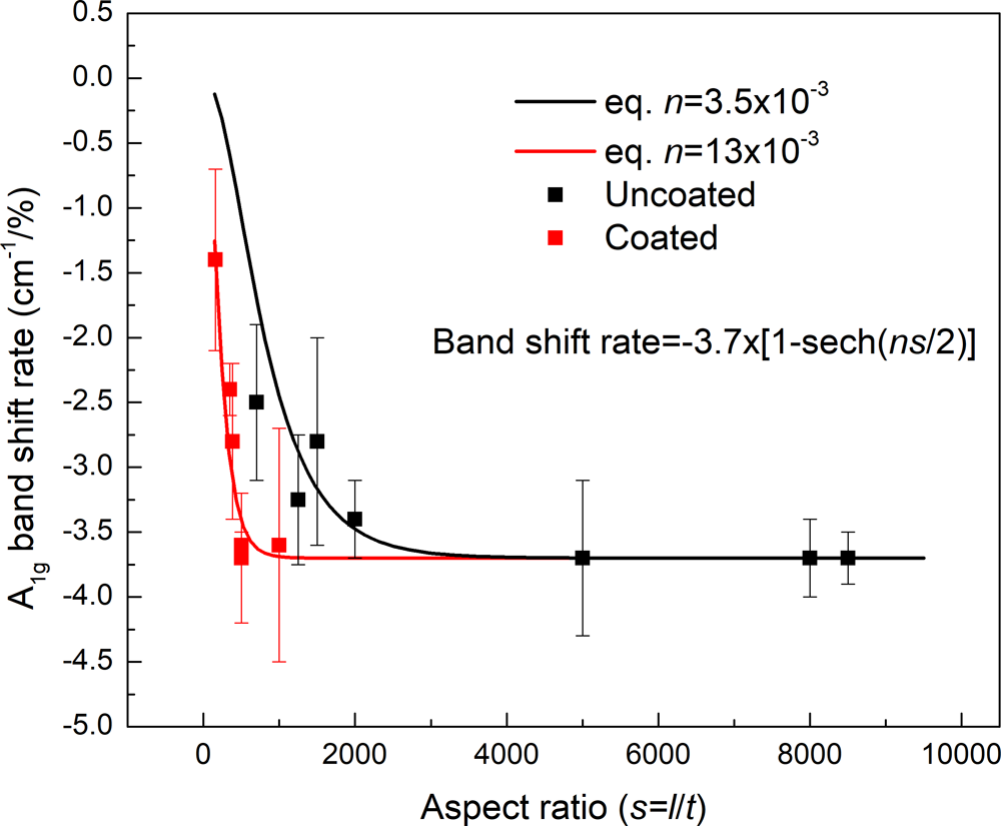
A_1g_ band shift rate against the aspect ratio of all the flakes measured at the flake center. The black and red data points are for the uncoated and coated flakes, respectively. The theoretical lines were plotted based on eq 5 with *n* values of 3.5 × 10^–3^ and 13 × 10^–3^, which fit well with uncoated and coated samples, respectively.

The values of the band shift rate measured at the center of the flakes are plotted as a function of the aspect ratio (*s*) of the flakes in Figure 5. It should be noted that the aspect ratio of the flakes was determined by taking the thickness of the flakes *t* = *t*_AFM_ – 2 as a calibration. Figure 5 shows that the values of band shift of uncoated flakes fit well with the theoretical curve (eq 5) with an *n* value of 3.5 × 10^–3^. This value shows excellent consistency with the monolayer flakes discussed in Figure 4. For the coated flakes, on the other hand, data points lie on the theoretical curve with an *n* value of 13 × 10^–3^, indicating a higher stress transfer efficiency from the matrix to the flakes due to the top coating of PMMA. The top-coated flakes can be analogous to the case when MXenes are mixed homogeneously in a polymer matrix, where the 2D material has two interfaces with the polymer. From Figure 5, we can conclude that the critical length for stress transfer of MXene can be reached when the aspect ratio of a coated flake is greater than ∼500.

We can then estimate the reinforcing efficiency of MXene flakes with various sizes in a matrix polymer using the rule-of-mixtures adapted for 2D materials, where the modulus of the composite is given by
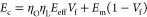
6where η_O_ is the orientation factor that is between 0.53 for random orientation and 1 for in-plane orientation, *E*_eff_ is the effective modulus of the flake, *V*_f_ is the volume fraction of the filler, and η_L_ is the length factor which is given by
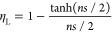
7The product (η_O_·η_L_·*E*_eff_) can be rewritten as *E*_f_, the filler modulus, taking the orientation and length factors into account.

We hereby analyze the reinforcing efficiency of multilayer flakes evaluated. The contour maps of A_1g_ band shift of a coated multilayer were obtained from Raman mapping and shown in Figure S5. The *ns* value was determined to be 8 for the flake with a thickness of >40 nm and a lateral size of 19 μm (aspect ratio ∼500). With respect to this multilayer, we can give the effective modulus of the flake under uniaxial tension using eq 7. The band shift rate is −3.7 cm^–1^/% (flake no. 5 in Table 1), the same as the monolayers measured. The value of *E*_eff_ can therefore take 330 GPa, while the *ns* value was evaluated to be 8 and η_L_ was calculated to be 0.75. For this flake, the filler modulus *E*_f_ (=η_O_·η_L_·*E*_eff_) is therefore determined to be ∼250 GPa for perfect in-plane orientation. In the case when loading of flakes with similar sizes dispersed in bulk composite samples, the filler modulus would reduce to ∼125 GPa for randomly oriented fillers. However, the control of flake size of MXene remains a challenge since the aspect ratio of a large batch of MXene flakes produced could vary significantly. We line mapped two more flakes with lower aspect ratios (flake nos. 4 and 7), as shown in Figure S6. The *ns* values obtained were around 5. Using the same approach, the filler modulus *E*_f_ (=η_O_·η_L_·*E*_eff_) is determined to be ∼170 GPa for perfectly in-plane orientation and ∼85 GPa for random orientation. This is similar order to the effective modulus estimated for a multilayer MXene in an epoxy matrix (22–66 GPa).^24^ The slight discrepancy could be due to the possible poor interface of some of the flakes (such as flake no. 6 in this work that shows no band shift under uniaxial strain) or varied morphologies (loops or folds as shown in Figure S1) in the bulk composite samples, which may give a little contribution to reinforcement.

## Conclusions

In this work, the mechanical properties of Ti_3_C_2_T*_x_* MXene on a polymer substrate were investigated by Raman spectroscopy, while a uniaxial strain was applied *in situ*. The thicknesses of the flakes on PMMA were examined using atomic force microscopy (AFM) and correlated with the Raman intensity ratio of *I*_MXene_/*I*_PMMA_ (A_1g_/C=O), allowing Raman spectroscopy to be used for estimating the flake thickness. Moreover, the interlayer and interfacial stress transfer of the MXene were investigated using strain-induced Raman band shifts by focusing on the A_1g_ band of Ti_3_C_2_T*_x_* at 730 cm^–1^. The average shift rate of the A_1g_ Raman band of MXene was determined to be ∼3.3 cm^–1^/% for strains up to 0.4–0.6%, giving a Grüneisen parameter of 0.70 ± 0.15. Importantly, it was shown that the band shift rate was much less dependent upon the number of layers than for multilayer graphene, indicating better interlayer stress transfer; hence, an MXene’s modulus is less dependent upon thickness. The strain along the MXene flakes was further mapped using Raman spectroscopy at different strains of the polymer substrate. It was demonstrated that the strain distributions across the MXene flakes on PMMA displayed an excellent agreement with the shear-lag theory. The interfacial shear strength at the flake ends was evaluated to be of the order of 3–4 MPa, up to 0.4% strain, *i.e*., similar to graphene,^29^ indicating that the polymer/MXene interfacial stress transfer took place principally through van der Waals bonding. The effective modulus of multilayer MXene, taking the size effect into account, was evaluated to be from the order of 10–100 GPa, depending on the aspect ratio of the flake. Overall, we have demonstrated that the most influential factor determining the mechanical performance of Ti_3_C_2_T*_x_* in a polymer matrix is the aspect ratio of the flake. An effective aspect ratio of the order of >500 is expected to facilitate good interfacial stress transfer and therefore give the highest effective modulus when used to reinforce polymers. This aspect ratio is currently achievable in 1–10 layer MXene flakes.

## Supporting Information Available

S1. Morphological characterization of MXene on PMMA substrates; S2. Strain-induced Raman band shift; and S3. Interfacial stress transfer of multilayers (PDF)

## References

[ref1] VahidMohammadi A.; Rosen J.; Gogotsi Y. The World of Two-dimensional Carbides and Nitrides (MXenes). Science 2021, 372, eabf1581 10.1126/science.abf1581. 34112665

[ref2] Gogotsi Y.; Huang Q. MXenes: Two-Dimensional Building Blocks for Future Materials and Devices. ACS Nano 2021, 15, 5775–5780. 10.1021/acsnano.1c03161. 33906288

[ref3] Zhou Y.; Maleski K.; Anasori B.; Thostenson J. O.; Pang Y.; Feng Y.; Zeng K.; Parker C. B.; Zauscher S.; Gogotsi Y.; Glass J. T.; Cao C. Ti_3_C_2_T_x_ MXene-Reduced Graphene Oxide Composite Electrodes for Stretchable Supercapacitors. ACS Nano 2020, 14, 3576–3586. 10.1021/acsnano.9b10066. 32049485

[ref4] Zhang Y.; Mu Z.; Lai J.; Chao Y.; Yang Y.; Zhou P.; Li Y.; Yang W.; Xia Z.; Guo S. MXene/Si@SiO_x_@C Layer-by-Layer Superstructure with Autoadjustable Function for Superior Stable Lithium Storage. ACS Nano 2019, 13, 2167–2175. 10.1021/acsnano.8b08821. 30689350

[ref5] Lyu B.; Kim M.; Jing H.; Kang J.; Qian C.; Lee S.; Cho J. H. Large-Area MXene Electrode Array for Flexible Electronics. ACS Nano 2019, 13, 11392–11400. 10.1021/acsnano.9b04731. 31553884

[ref6] Yao L.; Gu Q.; Yu X. Three-Dimensional MOFs@MXene Aerogel Composite Derived MXene Threaded Hollow Carbon Confined CoS Nanoparticles toward Advanced Alkali-Ion Batteries. ACS Nano 2021, 15, 3228–3240. 10.1021/acsnano.0c09898. 33508192

[ref7] Kurra N.; Uzun S.; Valurouthu G.; Gogotsi Y. Mapping (Pseudo)Capacitive Charge Storage Dynamics in Titanium Carbide MXene Electrodes in Aqueous Electrolytes Using 3D Bode Analysis. Energy Storage Mater. 2021, 39, 347–353. 10.1016/j.ensm.2021.04.037.

[ref8] Anasori B.; Lukatskaya M. R.; Gogotsi Y. 2D Metal Carbides and Nitrides (MXenes) for Energy Storage. Nat. Rev. Mater. 2017, 2, 16098 10.1038/natrevmats.2016.98.

[ref9] Naguib M.; Kurtoglu M.; Presser V.; Lu J.; Niu J.; Heon M.; Hultman L.; Gogotsi Y.; Barsoum M. W. Two-Dimensional Nanocrystals Produced by Exfoliation of Ti_3_AlC_2_. Adv. Mater. 2011, 23, 4248–4253. 10.1002/adma.201102306. 21861270

[ref10] Ling Z.; Ren C. E.; Zhao M.-Q.; Yang J.; Giammarco J. M.; Qiu J.; Barsoum M. W.; Gogotsi Y. Flexible and Conductive MXene Films and Nanocomposites with High Capacitance. Proc. Natl. Acad. Sci. 2014, 111, 16676 10.1073/pnas.1414215111. 25389310 PMC4250111

[ref11] Li X.; Yin X.; Liang S.; Li M.; Cheng L.; Zhang L. 2D Carbide MXene Ti_2_CT_x_ as a Novel High-performance Electromagnetic Interference Shielding Material. Carbon 2019, 146, 210–217. 10.1016/j.carbon.2019.02.003.

[ref12] Wan Y.-J.; Li X.-M.; Zhu P.-L.; Sun R.; Wong C.-P.; Liao W.-H. Lightweight, Flexible MXene/polymer Film with Simultaneously Excellent Mechanical Property and High-performance Electromagnetic Interference Shielding. Composites, Part A 2020, 130, 105764 10.1016/j.compositesa.2020.105764.

[ref13] Liu R.; Miao M.; Li Y.; Zhang J.; Cao S.; Feng X. Ultrathin Biomimetic Polymeric Ti_3_C_2_T_x_ MXene Composite Films for Electromagnetic Interference Shielding. ACS Appl. Mater. Interfaces 2018, 10, 44787–44795. 10.1021/acsami.8b18347. 30516359

[ref14] Tu S.; Jiang Q.; Zhang J.; He X.; Hedhili M. N.; Zhang X.; Alshareef H. N. Enhancement of Dielectric Permittivity of Ti_3_C_2_T_x_ MXene/Polymer Composites by Controlling Flake Size and Surface Termination. ACS Appl. Mater. Interfaces 2019, 11, 27358–27362. 10.1021/acsami.9b09137. 31305992

[ref15] Tu S.; Jiang Q.; Zhang X.; Alshareef H. N. Large Dielectric Constant Enhancement in MXene Percolative Polymer Composites. ACS Nano 2018, 12, 3369–3377. 10.1021/acsnano.7b08895. 29624367

[ref16] Li L.; Fu X.; Chen S.; Uzun S.; Levitt A. S.; Shuck C. E.; Han W.; Gogotsi Y. Hydrophobic and Stable MXene–Polymer Pressure Sensors for Wearable Electronics. ACS Appl. Mater. Interfaces 2020, 12, 15362–15369. 10.1021/acsami.0c00255. 32159323

[ref17] Lee S.; Kim E. H.; Yu S.; Kim H.; Park C.; Park T. H.; Han H.; Lee S. W.; Baek S.; Jin W.; Koo C. M.; Park C. Alternating-Current MXene Polymer Light-Emitting Diodes. Adv. Funct. Mater. 2020, 30, 2001224 10.1002/adfm.202070212.

[ref18] Malaki M.; Varma R. S. Mechanotribological Aspects of MXene-Reinforced Nanocomposites. Adv. Mater. 2020, 32, 2003154 10.1002/adma.202003154. 32779252

[ref19] Zhang H.; Wang L.; Chen Q.; Li P.; Zhou A.; Cao X.; Hu Q. Preparation, Mechanical and Anti-friction Performance of MXene/polymer Composites. Mater. Des. 2016, 92, 682–689. 10.1016/j.matdes.2015.12.084.

[ref20] Boota M.; Gogotsi Y. MXene—Conducting Polymer Asymmetric Pseudocapacitors. Adv. Energy Mater. 2019, 9, 1802917 10.1002/aenm.201802917.

[ref21] Li J.; Levitt A.; Kurra N.; Juan K.; Noriega N.; Xiao X.; Wang X.; Wang H.; Alshareef H. N.; Gogotsi Y. MXene-conducting Polymer Electrochromic Microsupercapacitors. Energy Storage Mater. 2019, 20, 455–461. 10.1016/j.ensm.2019.04.028.

[ref22] Gund G. S.; Park J. H.; Harpalsinh R.; Kota M.; Shin J. H.; Kim T.-i.; Gogotsi Y.; Park H. S. MXene/Polymer Hybrid Materials for Flexible AC-Filtering Electrochemical Capacitors. Joule 2019, 3, 164–176. 10.1016/j.joule.2018.10.017.

[ref23] Boota M.; Anasori B.; Voigt C.; Zhao M.-Q.; Barsoum M. W.; Gogotsi Y. Pseudocapacitive Electrodes Produced by Oxidant-Free Polymerization of Pyrrole between the Layers of 2D Titanium Carbide (MXene). Adv. Mater. 2016, 28, 1517–1522. 10.1002/adma.201504705. 26660424

[ref24] Monastyreckis G.; Mishnaevsky L.; Hatter C. B.; Aniskevich A.; Gogotsi Y.; Zeleniakiene D. Micromechanical Modeling of MXene-polymer Composites. Carbon 2020, 162, 402–409. 10.1016/j.carbon.2020.02.070.

[ref25] Lipatov A.; Lu H.; Alhabeb M.; Anasori B.; Gruverman A.; Gogotsi Y.; Sinitskii A. Elastic Properties of 2D Ti_3_C_2_T_x_ MXene Monolayers and Bilayers. Sci. Adv. 2018, 4, eaat0491 10.1126/sciadv.aat0491. 29922719 PMC6003751

[ref26] Firestein K. L.; von Treifeldt J. E.; Kvashnin D. G.; Fernando J. F. S.; Zhang C.; Kvashnin A. G.; Podryabinkin E. V.; Shapeev A. V.; Siriwardena D. P.; Sorokin P. B.; Golberg D. Young’s Modulus and Tensile Strength of Ti_3_C_2_ MXene Nanosheets As Revealed by In Situ TEM Probing, AFM Nanomechanical Mapping, and Theoretical Calculations. Nano Lett. 2020, 20, 5900–5908. 10.1021/acs.nanolett.0c01861. 32633975

[ref27] Young R. J.; Gong L.; Kinloch I. A.; Riaz I.; Jalil R.; Novoselov K. S. Strain Mapping in a Graphene Monolayer Nanocomposite. ACS Nano 2011, 5, 3079–3084. 10.1021/nn2002079. 21395299

[ref28] Gong L.; Young R. J.; Kinloch I. A.; Riaz I.; Jalil R.; Novoselov K. S. Optimizing the Reinforcement of Polymer-Based Nanocomposites by Graphene. ACS Nano 2012, 6, 2086–2095. 10.1021/nn203917d. 22364317

[ref29] Gong L.; Kinloch I. A.; Young R. J.; Riaz I.; Jalil R.; Novoselov K. S. Interfacial Stress Transfer in a Graphene Monolayer Nanocomposite. Adv. Mater. 2010, 22, 2694–2697. 10.1002/adma.200904264. 20473982

[ref30] Young R. J.; Liu M.; Kinloch I. A.; Li S.; Zhao X.; Vallés C.; Papageorgiou D. G. The Mechanics of Reinforcement of Polymers by Graphene Nanoplatelets. Compos. Sci. Technol. 2018, 154, 110–116. 10.1016/j.compscitech.2017.11.007.

[ref31] Zhao X.; Papageorgiou D. G.; Zhu L.; Ding F.; Young R. J. The Strength of Mechanically-exfoliated Monolayer Graphene Deformed on a Rigid Polymer Substrate. Nanoscale 2019, 11, 14339–14353. 10.1039/C9NR04720D. 31328739

[ref32] Li Z.; Young R. J.; Papageorgiou D. G.; Kinloch I. A.; Zhao X.; Yang C.; Hao S. Interfacial Stress Transfer in Strain Engineered Wrinkled and Folded Graphene. 2D Mater. 2019, 6, 045026 10.1088/2053-1583/ab3167.

[ref33] Liu M.; Li Z.; Zhao X.; Young R. J.; Kinloch I. A. Fundamental Insights into Graphene Strain Sensing. Nano Lett. 2021, 21, 833–839. 10.1021/acs.nanolett.0c04577. 33372510

[ref34] Li Z.; Kinloch I. A.; Young R. J.; Novoselov K. S.; Anagnostopoulos G.; Parthenios J.; Galiotis C.; Papagelis K.; Lu C.-Y.; Britnell L. Deformation of Wrinkled Graphene. ACS Nano 2015, 9, 3917–3925. 10.1021/nn507202c. 25765609 PMC4424820

[ref35] Frank O.; Mohr M.; Maultzsch J.; Thomsen C.; Riaz I.; Jalil R.; Novoselov K. S.; Tsoukleri G.; Parthenios J.; Papagelis K.; Kavan L.; Galiotis C. Raman 2D-Band Splitting in Graphene: Theory and Experiment. ACS Nano 2011, 5, 2231–2239. 10.1021/nn103493g. 21319849

[ref36] Androulidakis C.; Koukaras E. N.; Paterakis G.; Trakakis G.; Galiotis C. Tunable Macroscale Structural Superlubricity in Two-layer Graphene via Strain Engineering. Nat. Commun. 2020, 11, 1595 10.1038/s41467-020-15446-y. 32221301 PMC7101365

[ref37] Pastore Carbone M. G.; Manikas A. C.; Souli I.; Pavlou C.; Galiotis C. Mosaic Pattern Formation in Exfoliated Graphene by Mechanical Deformation. Nat. Commun. 2019, 10, 1572 10.1038/s41467-019-09489-z. 30952849 PMC6450902

[ref38] Mohiuddin T. M. G.; Lombardo A.; Nair R. R.; Bonetti A.; Savini G.; Jalil R.; Bonini N.; Basko D. M.; Galiotis C.; Marzari N.; Novoselov K. S.; Geim A. K.; Ferrari A. C. Uniaxial Strain in Graphene by Raman Spectroscopy: *G* Peak Splitting, Grüneisen Parameters, and Sample Orientation. Phys. Rev. B 2009, 79, 205433 10.1103/PhysRevB.79.205433.

[ref39] De Sanctis A.; Mehew J. D.; Alkhalifa S.; Withers F.; Craciun M. F.; Russo S. Strain-Engineering of Twist-Angle in Graphene/hBN Superlattice Devices. Nano Lett. 2018, 18, 7919–7926. 10.1021/acs.nanolett.8b03854. 30474986

[ref40] Wang F.; Kinloch I. A.; Wolverson D.; Tenne R.; Zak A.; O’Connell E.; Bangert U.; Young R. J. Strain-induced Phonon Shifts in Tungsten Disulfide Nanoplatelets and Nanotubes. 2D Mater. 2017, 4, 015007 10.1088/2053-1583/4/1/015007.

[ref41] Wang F.; Li S.; Bissett M. A.; Kinloch I. A.; Li Z.; Young R. J. Strain Engineering in Monolayer WS_2_ and WS_2_ Nanocomposites. 2D Mater. 2020, 7, 045022 10.1088/2053-1583/ababf1.

[ref42] Dadgar A. M.; Scullion D.; Kang K.; Esposito D.; Yang E. H.; Herman I. P.; Pimenta M. A.; Santos E. J. G.; Pasupathy A. N. Strain Engineering and Raman Spectroscopy of Monolayer Transition Metal Dichalcogenides. Chem. Mater. 2018, 30, 5148–5155. 10.1021/acs.chemmater.8b01672.

[ref43] Dai Z.; Liu L.; Zhang Z. Strain Engineering of 2D Materials: Issues and Opportunities at the Interface. Adv. Mater. 2019, 31, 1805417 10.1002/adma.201805417. 30650204

[ref44] Wang W.; Li Z.; Marsden A.; Bissett M. A.; Young R. J. Interlayer and Interfacial Stress Transfer in hBN Nanosheets. 2D Mater. 2021, 8, 035058 10.1088/2053-1583/ac0c2a.

[ref45] Androulidakis C.; Koukaras E. N.; Poss M.; Papagelis K.; Galiotis C.; Tawfick S. Strained Hexagonal Boron Nitride: Phonon Shift and Grüneisen Parameter. Phys. Rev. B 2018, 97, 241414 10.1103/PhysRevB.97.241414.

[ref46] Seremetis L.; Koukaras E. N.; Alexandri S.; Michail A.; Kalosakas G.; Parthenios J.; Galiotis C.; Tsirkas S.; Grammatikopoulos S.; Papagelis K. Thermomechanical Response of Supported Hexagonal Boron Nitride Sheets of Various Thicknesses. J. Phys. Chem. C 2020, 124, 12134–12143. 10.1021/acs.jpcc.0c01029.

[ref47] Wang L.; Zihlmann S.; Baumgartner A.; Overbeck J.; Watanabe K.; Taniguchi T.; Makk P.; Schönenberger C. In Situ Strain Tuning in hBN-Encapsulated Graphene Electronic Devices. Nano Lett. 2019, 19, 4097–4102. 10.1021/acs.nanolett.9b01491. 31117761

[ref48] Mathis T. S.; Maleski K.; Goad A.; Sarycheva A.; Anayee M.; Foucher A. C.; Hantanasirisakul K.; Shuck C. E.; Stach E. A.; Gogotsi Y. Modified MAX Phase Synthesis for Environmentally Stable and Highly Conductive Ti_3_C_2_ MXene. ACS Nano 2021, 15, 6420–6429. 10.1021/acsnano.0c08357. 33848136

[ref49] Lipatov A.; Alhabeb M.; Lukatskaya M. R.; Boson A.; Gogotsi Y.; Sinitskii A. Effect of Synthesis on Quality, Electronic Properties and Environmental Stability of Individual Monolayer Ti_3_C_2_ MXene Flakes. Adv. Electron. Mater. 2016, 2, 1600255 10.1002/aelm.201670068.

[ref50] Ghidiu M.; Lukatskaya M. R.; Zhao M.-Q.; Gogotsi Y.; Barsoum M. W. Conductive Two-dimensional Titanium Carbide ‘Clay’ with High Volumetric Capacitance. Nature 2014, 516, 78–81. 10.1038/nature13970. 25470044

[ref51] Wang X.; Shen X.; Gao Y.; Wang Z.; Yu R.; Chen L. Atomic-Scale Recognition of Surface Structure and Intercalation Mechanism of Ti_3_C_2_X. J. Am. Chem. Soc. 2015, 137, 2715–2721. 10.1021/ja512820k. 25688582

[ref52] Novoselov K. S.; Geim A. K.; Morozov S. V.; Jiang D.; Katsnelson M. I.; Grigorieva I. V.; Dubonos S. V.; Firsov A. A. Two-dimensional Gas of Massless Dirac Fermions in Graphene. Nature 2005, 438, 197–200. 10.1038/nature04233. 16281030

[ref53] Xu K.; Cao P.; Heath J. R. Graphene Visualizes the First Water Adlayers on Mica at Ambient Conditions. Science 2010, 329, 1188 10.1126/science.1192907. 20813950

[ref54] Ochedowski O.; Bussmann B. K.; Schleberger M. Graphene on Mica - Intercalated Water Trapped for Life. Sci. Rep. 2015, 4, 6003 10.1038/srep06003. PMC4135328 25132493

[ref55] Coy Diaz H.; Addou R.; Batzill M. Interface Properties of CVD Grown Graphene Transferred onto MoS_2_ (0001). Nanoscale 2014, 6, 1071–1078. 10.1039/C3NR03692H. 24297086

[ref56] Sarycheva A.; Gogotsi Y. Raman Spectroscopy Analysis of the Structure and Surface Chemistry of Ti_3_C_2_T_x_ MXene. Chem. Mater. 2020, 32, 3480–3488. 10.1021/acs.chemmater.0c00359.

[ref57] Hu T.; Wang J.; Zhang H.; Li Z.; Hu M.; Wang X. Vibrational Properties of Ti_3_C_2_ and Ti_3_C_2_T_2_ (T=O, F, OH) Monosheets by First-principles Calculations: a Comparative Study. Phys. Chem. Chem. Phys. 2015, 17, 9997–10003. 10.1039/C4CP05666C. 25785395

[ref58] Hu C.; Chen X.; Chen J.; Zhang W.; Zhang M. Q. Observation of Mutual Diffusion of Macromolecules in PS/PMMA Binary Films by Confocal Raman Microscopy. Soft Matter 2012, 8, 4780–4787. 10.1039/C2SM07299H.

[ref59] Rice C.; Young R. J.; Zan R.; Bangert U.; Wolverson D.; Georgiou T.; Jalil R.; Novoselov K. S. Raman-scattering Measurements and First-principles Calculations of Strain-induced Phonon Shifts in Monolayer MoS_2_. Phys. Rev. B 2013, 87, 081307 10.1103/PhysRevB.87.081307.

[ref60] Zhang L.; Su W.; Huang Y.; Li H.; Fu L.; Song K.; Huang X.; Yu J.; Lin C.-T. In Situ High-Pressure X-ray Diffraction and Raman Spectroscopy Study of Ti_3_C_2_T_x_ MXene. Nanoscale Res. Lett. 2018, 13, 343 10.1186/s11671-018-2746-4. 30374742 PMC6206307

[ref61] Munn R. W. Grüneisen Parameters for Molecular Crystals. Phys. Rev. B 1975, 12, 3491–3493. 10.1103/PhysRevB.12.3491.

[ref62] Hanfland M.; Beister H.; Syassen K. Graphite Under Pressure: Equation of State and first-order Raman Modes. Phys. Rev. B 1989, 39, 12598–12603. 10.1103/PhysRevB.39.12598. 9948126

[ref63] Mu M.; Osswald S.; Gogotsi Y.; Winey K. I. An *in situ* Raman Spectroscopy Study of Stress Transfer between Carbon Nanotubes and Polymer. Nanotechnology 2009, 20, 335703 10.1088/0957-4484/20/33/335703. 19636105

