# Supplementary material for: Deformation of and Interfacial Stress Transfer in Ti3C2 MXene–Polymer Composites
Source: ACS Appl Mater Interfaces. 2022 Feb 21;14(8):10681–90. doi: 10.1021/acsami.1c21611 (PMC9171720; doi:10.1021/acsami.1c21611)
Supplement: Supplementary file 1 — am1c21611_si_001.pdf [file am1c21611_si_001.pdf]

Deformation of and Interfacial Stress Transfer in Ti_3C_2 MXene-Polymer Composites

Supporting information

*Mufeng Liu[1], Yuling Zhuo[1], Asia Sarycheva[2], Yury Gogotsi[2], Mark A. Bissett[1], Robert J. Young[1], Ian A. Kinloch[1] **

[1]National Graphene Institute, Henry Royce Institute and Department of Materials, School of Natural Sciences, The University of Manchester, Oxford Road, Manchester M13 9PL, UK

[2]A. J. Drexel Nanomaterials Institute, and Department of Materials Science and Engineering, Drexel University, Philadelphia, Pennsylvania 19104, United States

S1. Morphological characterization of MXene on PMMA substrates.

Typical morphologies of the MXene flakes deposited on the PMMA beams were examined using AFM. The AFM images of random MXene flakes are displayed Figure S1(a) and (b), while (c) and (d) are the measured thicknesses across the designated lines in (a) and (b) respectively. Overall, it can be seen that the MXene flakes can be folded which creates variable thicknesses across the whole flake, such as the thinner flake located at top-left corner in (a). In addition, some small and thicker areas can be found, possibly due to incomplete exfoliation or some small MXene flakes adhered to larger flakes (e.g. Figure S1b).

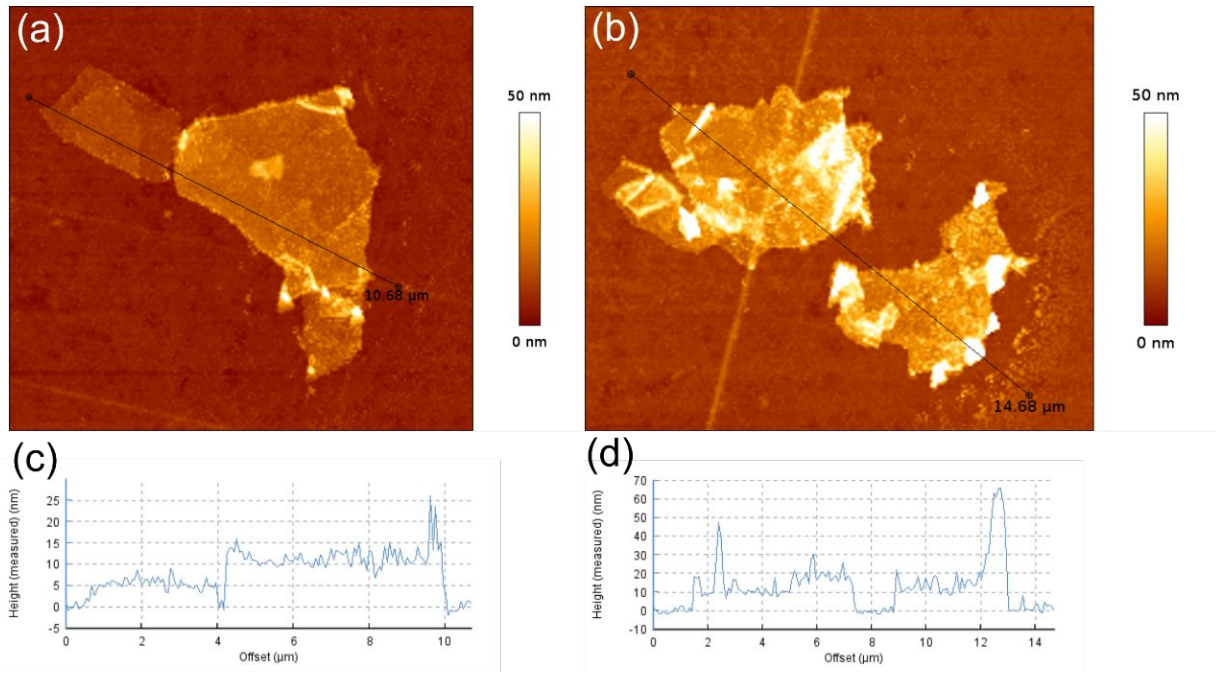

Figure S1. Representative morphologies of MXene on PMMA (a, b) AFM image of MXene flakes and (c, d) the height measured along the designated lines in (a) and (b) respectively.

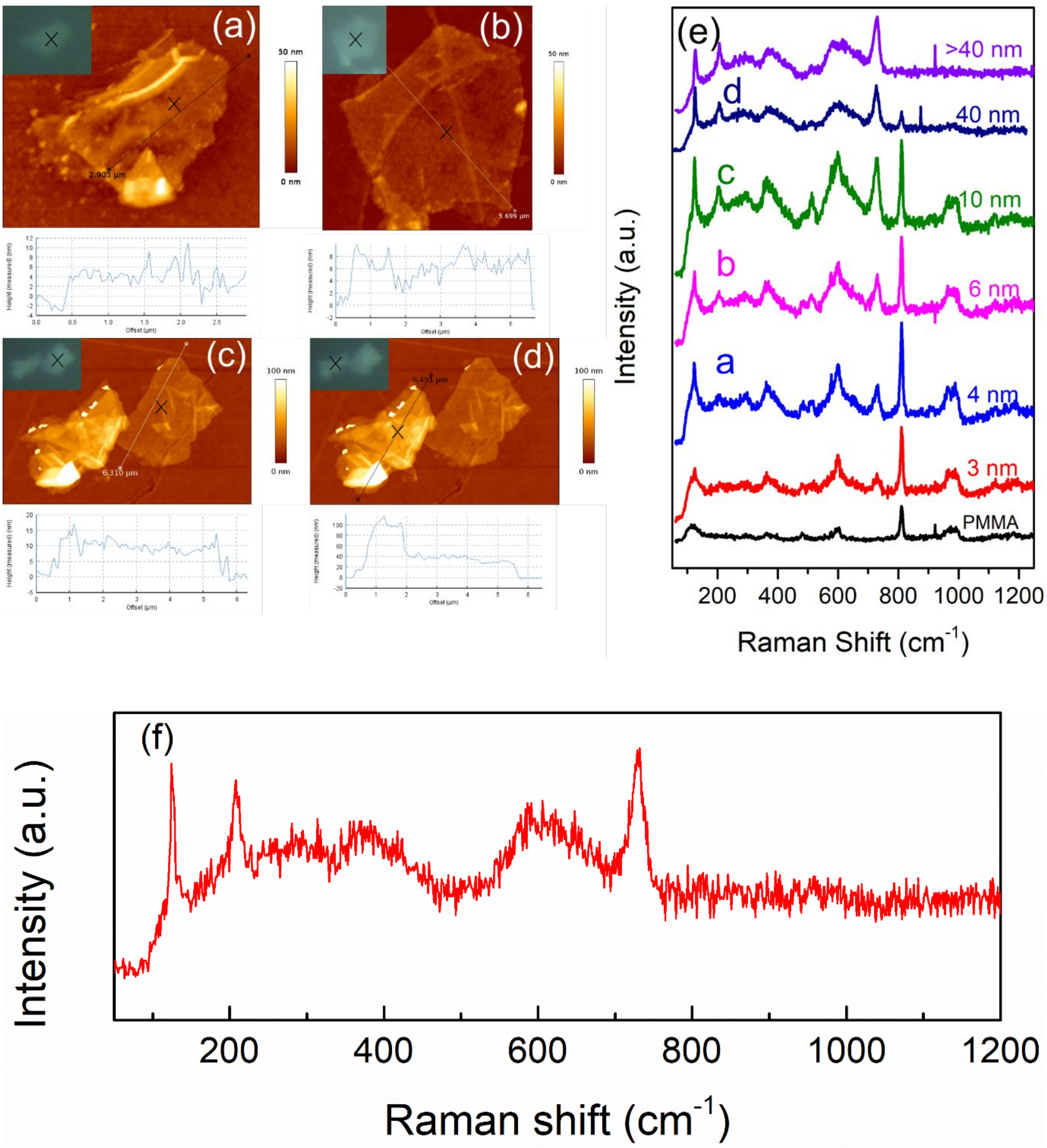

Figure S2. (a-d) The flakes that were measured by both AFM and Raman spectroscopy. The insets displayed the corresponding optical micrographs of each flake. The AFM height profiles were marked in the images and shown below each image. The cross signs (×) marked the location of the focus of the laser when the Raman spectra were taken which can be seen in both AFM and optical images. (e) Raman spectra of the flakes with different thicknesses; the red spectrum (~3 nm) is the flake in Figure 1(a) main text; Spectra (a-d) refer to Figure S2(a-d). (f) Raman spectrum of flake no.5. The flake no. 5 was top-coated with a thin layer of PMMA as

described in the section upon Experimental Methods. It can be seen that the signature peak of the PMMA at 810 cm^{-1} was not present, due to the very low thickness of the PMMA. This enabled us to estimate the thickness of the flakes for the top-coated samples.

S2. Strain induced Raman band shift

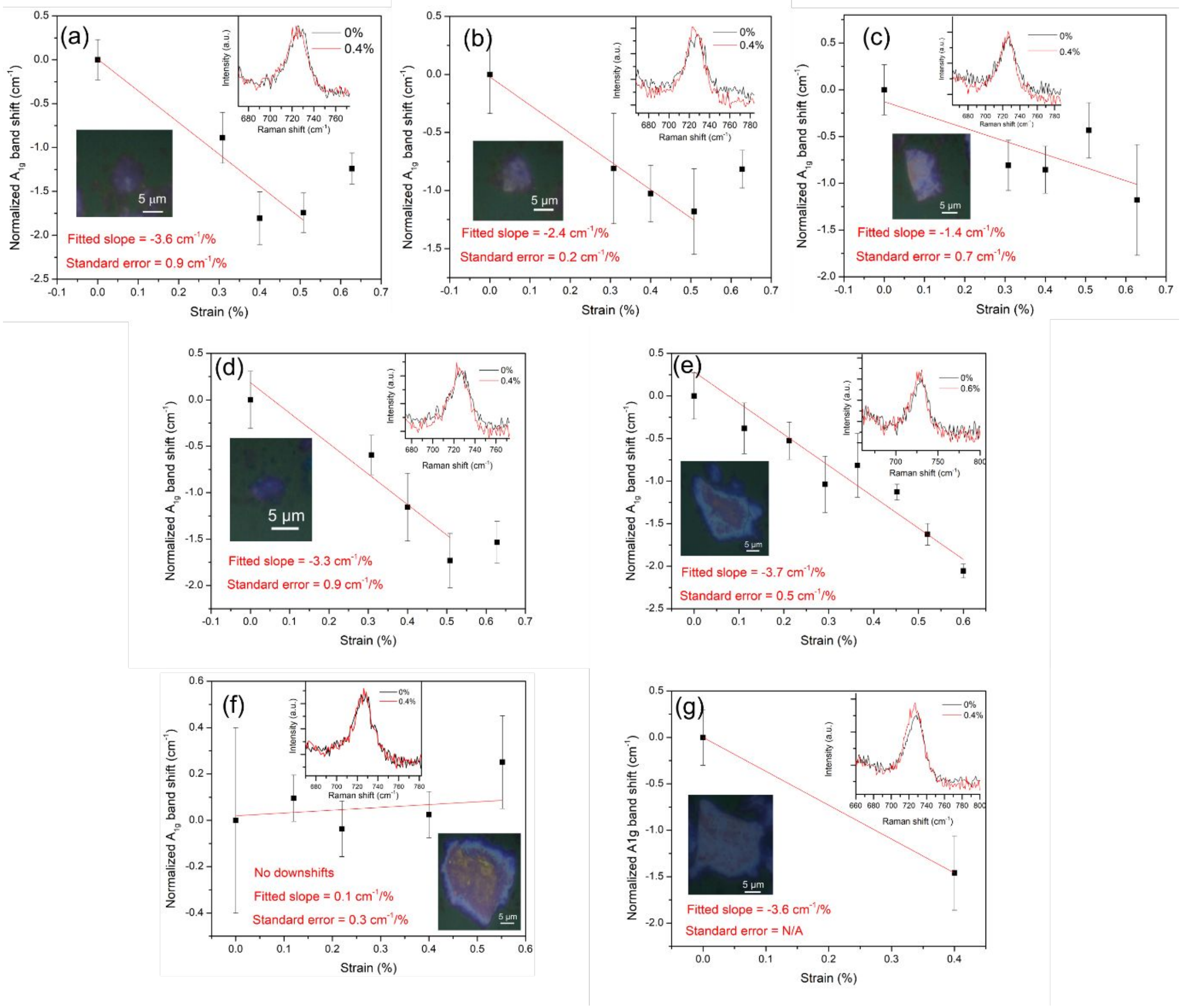

Figure S3. (a-g) Raman band shift measurements at the center of the flakes of no.1-7 in Table 1. The samples were top-coated with PMMA. The error bars were given by 5 times of repeated tests on the same point for each measurement. The standard error was given by instrumental weighing of the error bars. For (a-d), the linear fitting did not include the 0.6%

data as the Raman band started to show an upshift as a result of interfacial slipping at higher strains. In (g), the Raman band shift experiment was carried out by increasing the strain from 0% to 0.4% directly rather than gradually to double check the response of the strain induced Raman band shift. The standard error was not given by instrumental weighing of the error as there were only 2 data points.

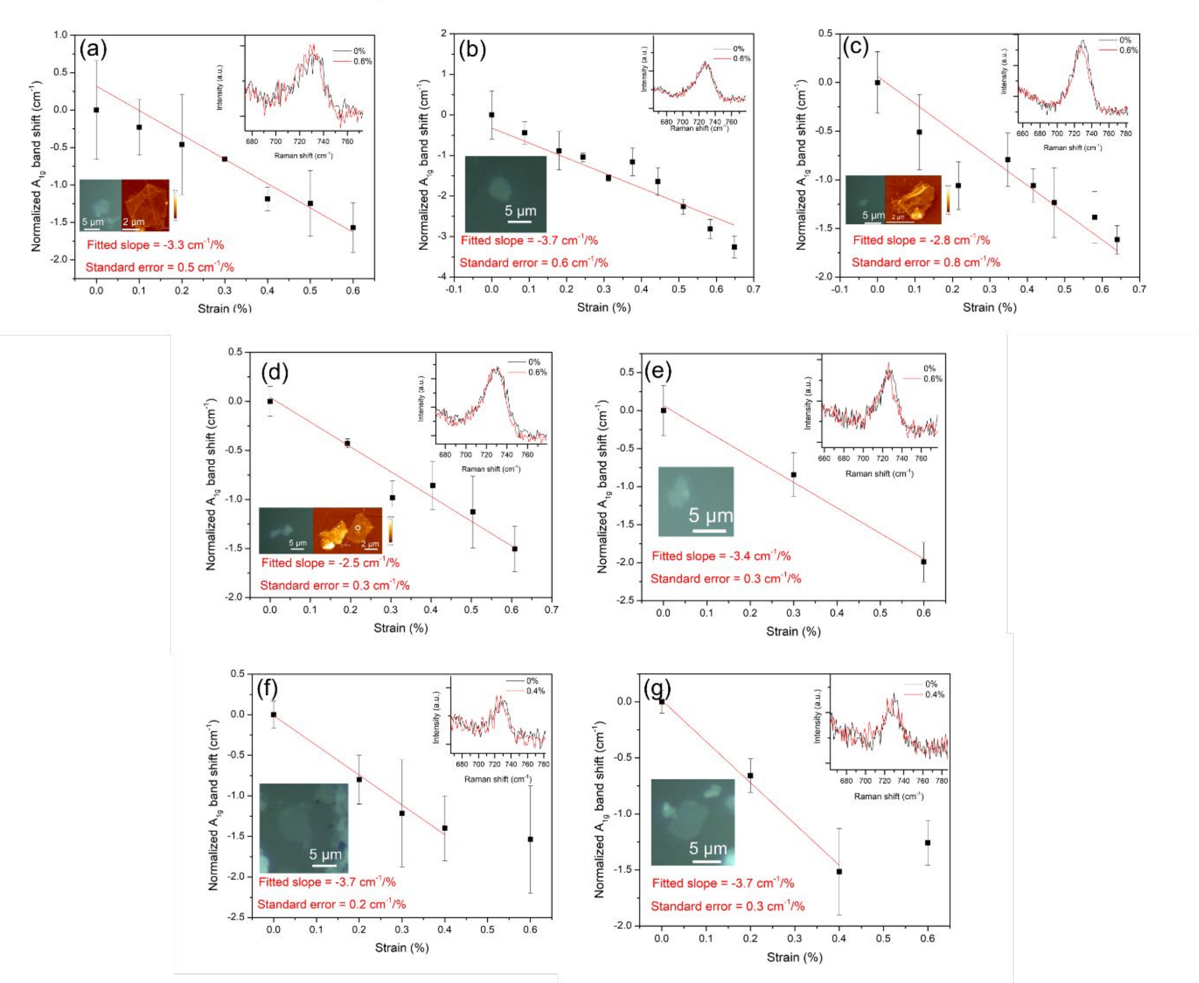

Figure S4. (a-g) Raman band shift measurements at the centers of the flakes of no.8-14 in Table 1. The samples were not coated with PMMA on the top. For (d), the measurement took place at the flake highlighted with a circle symbol. The error bars are given for 5 repeated tests on the same point for each measurement. The standard error was given by instrumental weighing of the error bars. For (f), the linear fitting did not include 0.6% strain data point as interfacial slippage occurred, which was confirmed by the Raman mapping in Figure 3. For

(g), the linear fitting did not include the 0.6% data point as the Raman band started to show an upshift as a result of interfacial slipping at 0.6% strain.

Table S1. Full width at half maximum (FWHM) of all the flakes measured.

Flake No.	1	2	3	4	5	6	7
FWHM (cm^{-1})	19.3 ± 1.3	19.5 ± 1.2	20.1 ± 0.8	19.1 ± 0.9	20.2 ± 1.3	18.5 ± 1.6	19.9 ± 1.2
Flake No.	8	9	10	11	12	13	14
FWHM (cm^{-1})	20.2 ± 1.2	19.8 ± 0.9	19.7 ± 0.8	20.9 ± 0.7	19.3 ± 1.2	20.4 ± 0.2	20.4 ± 1.3

S3. Interfacial stress transfer of multilayers

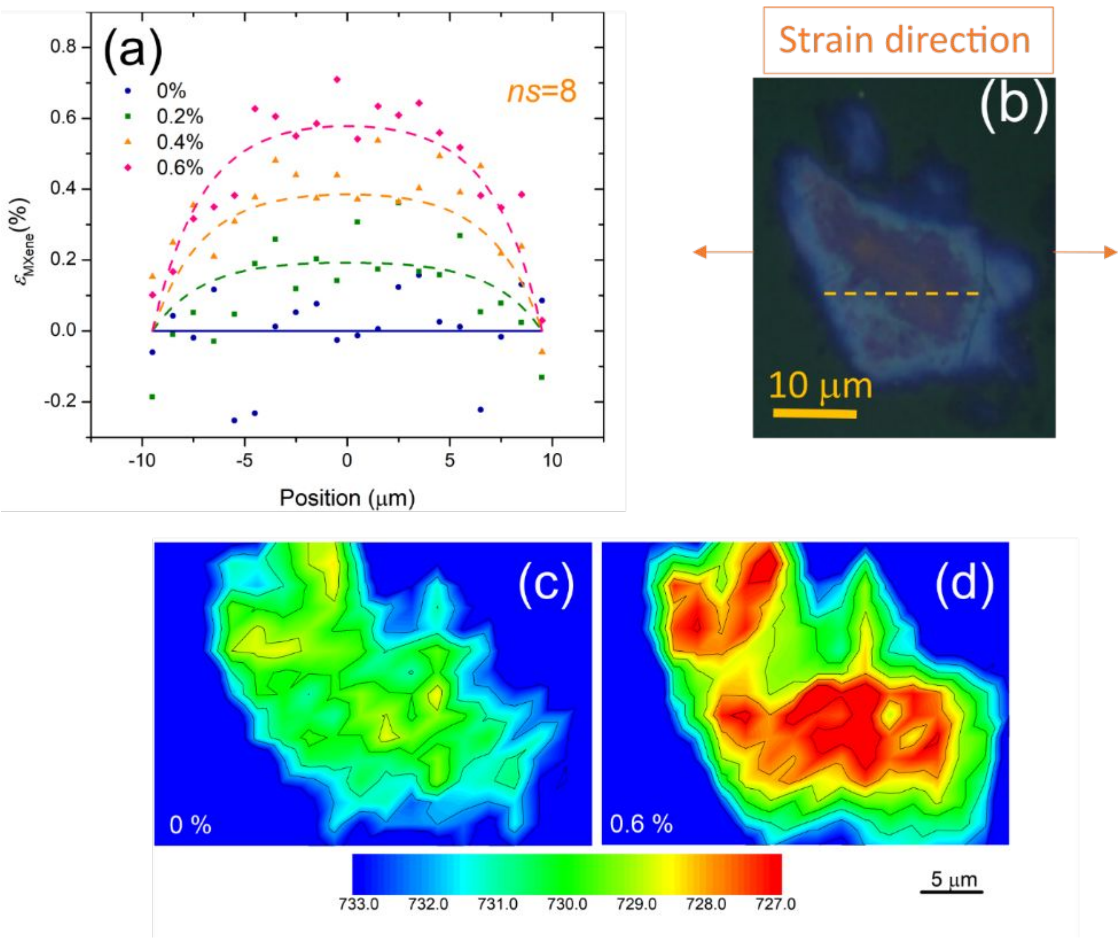

Figure S5. (a) Strain distribution of a multilayer MXene (>40 nm) at 0, 0.2, 0.4, 0.6% strains of the substrate; the sample was coated with a thin top layer of PMMA; The strain distributions were fitted using shear-lag theory; (b) Optical micrograph of the flake tested; the dashed line indicates position of the line mapping showing in (a), while the arrows indicate the strain direction (bending direction); (c) and (d) are the 2D mapping at the strains of 0% and 0.6% showing a representative strain distributions. This flake is flake no. 5 in **Table 1**.

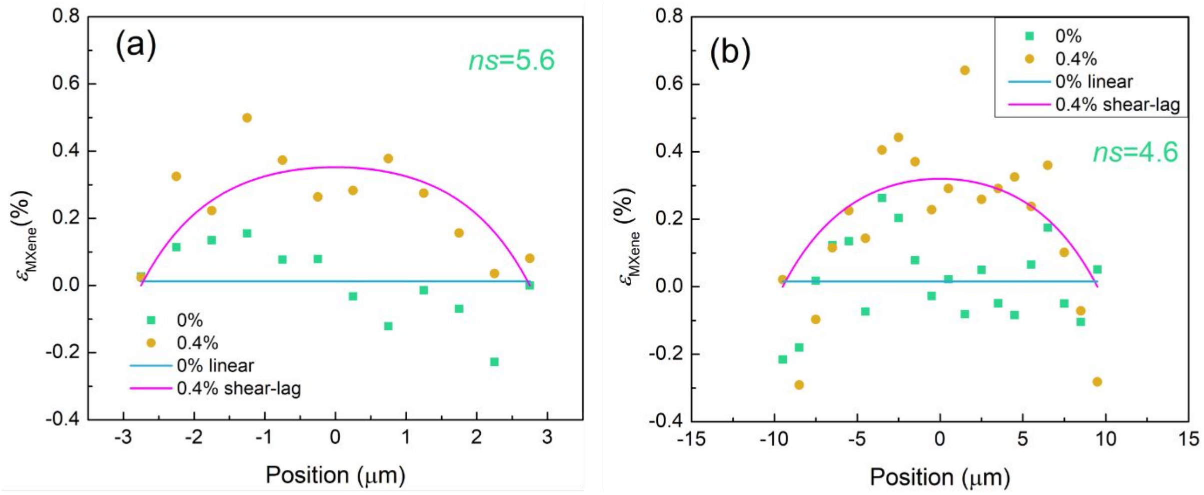

Figure S6. (a) flake No.7 and (b) flake No. 4 line mappings with shear-lag fittings, giving *ns* values 5.6 and 4.6, respectively. The data points show a significant scatter as a result of the low rate of band shift.
